# The Rice E3 Ubiquitin Ligase Gene *OsPUB77* Regulates Head Milled Rice Rate by Affecting Grain Starch Accumulation

**DOI:** 10.1111/pbi.70603

**Published:** 2026-02-19

**Authors:** Shuai Nie, Luo Chen, Leilei Kong, Minhua Zheng, Jingfang Dong, Song Bai, Dilin Liu, Shaohong Zhang, Hao Chen, Haifei Hu, Xin Luan, Junliang Zhao, Wu Yang

**Affiliations:** ^1^ Rice Research Institute, Guangdong Academy of Agricultural Sciences Guangzhou P. R. China; ^2^ Guangdong Key Laboratory of New Technology in Rice Breeding Guangzhou P. R. China; ^3^ Guangdong Rice Engineering Laboratory Guangzhou P. R. China; ^4^ Key Laboratory of Genetics and Breeding of High Quality Rice in Southern China (Co‐Construction by Ministry and Province), Ministry of Agriculture and Rural Affairs Guangzhou P. R. China

## Abstract

Head milled rice rate (HMRR) is a critical trait determining rice yield and economic value, yet its genetic basis remains poorly understood. Here, through genome‐wide association study across multiple environments, we identified a major QTL, qHMRR4‐2, and pinpointed OsPUB77, a U‐box E3 ubiquitin ligase, as the causal gene. Haplotype analysis and transgenic validation revealed that both knockout and overexpression of OsPUB77 significantly reduce HMRR by disrupting starch accumulation and increasing grain chalkiness. We further demonstrated that OsMADS29 directly binds to the OsPUB77 promoter and represses its transcription. Transcriptomic analysis indicated that OsPUB77 maintains metabolic homeostasis essential for starch biosynthesis during grain filling. Our findings establish the OsMADS29‐OsPUB77 module as a critical regulator of HMRR and provide a promising target for improving rice milling quality through precision breeding.

Head milled rice rate (HMRR), defined as the proportion of milled kernels retaining at least 75% of their original length, is a key determinant of rice productivity and market value (Zhu et al. 2024). Despite its importance, the genetic basis of this complex quantitative trait remains largely unclear, with only a few genes having been identified to date (Sreenivasulu 2019; Deng et al. 2022). This limited understanding hampers molecular breeding efforts aimed at improving this economically important trait.

To dissect the genetic architecture of HMRR, we evaluated the HMRR of 300 diverse *indica* accessions across two environments, identifying 15 associated QTLs via genome‐wide association study (GWAS) (Figure 1a; Figure S1, Tables S1 and S2). Four QTLs were co‐localized with previously reported QTLs or genes, and among the novel QTLs, *qHMRR4‐2* on chromosome 4 was consistently detected across two environments (Table S2). Within this locus, HMRR varied significantly among accessions carrying contrasting haplotypes (Figure 1b). Linkage disequilibrium decay analysis delimited *qHMRR4‐2* to a 260‐kb interval containing 31 annotated genes (Figure 1c and Table S3).

**FIGURE 1:**
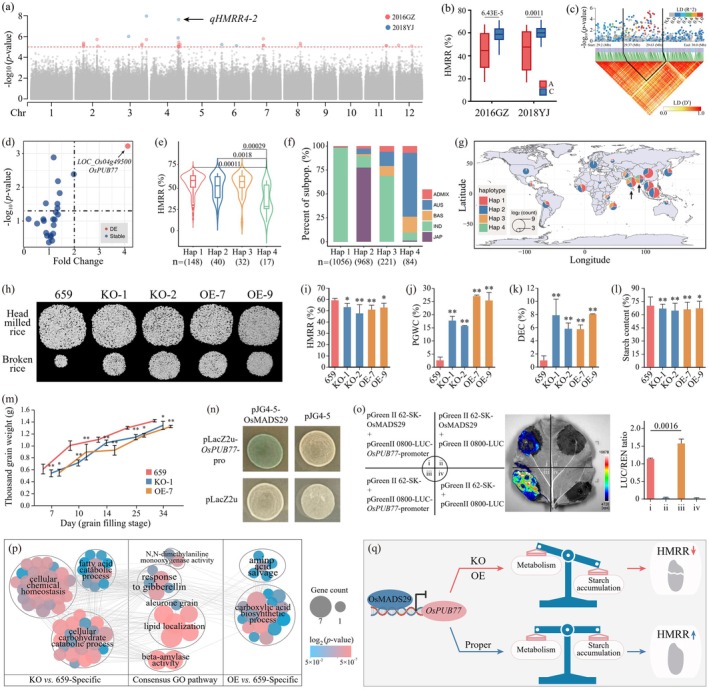
*OsPUB77* regulates head milled rice rate (HMRR) by affecting grain starch accumulation. (a) GWAS for HMRR. The 2016GZ and 2018YJ represent the two environments, respectively. (b) Phenotypic comparison between accessions corresponding to each of the two alleles of leading SNP (major allele ‘C’ vs. minor allele ‘A’ of Chr4‐29 582 749) for *qHMRR4‐2*. (c) The local Manhattan plot and the linkage disequilibrium heatmap for *qHMRR4‐2*. (d) In the LD block of *qHMRR4‐2*, a gene with significant differential expression (DE) was identified between samples corresponding to each of the two alleles (‘C’ vs. ‘A’) of leading SNP. Red dot was defined as DE gene, while blue dots represent stably expressed genes. (e) Comparison of HMRR among accessions harbouring four haplotypes of *OsPUB77*. (f) and (g) represent the population and geographic distributions of the four haplotypes among 3 K rice genomes, respectively. (h–m) Phenotypic comparisons among the wild type (an *indica* accession No. 659), gene knockout lines (KO‐1, KO‐2), and gene overexpression lines (OE‐7 and OE‐9) of *OsPUB77*. Values are means ± SD. Student's *t*‐test: *p < 0.05; **p < 0.01; ns, no significance. (h) Phenotypes of HMRR in wild type and transgenic lines. (i–m) represent the statistical results of HMRR, percentage of grains with chalkiness (PGWC), degree of endosperm chalkiness (DEC), starch content, and thousand grain weight of brown rice, respectively. (n) Y1H assay showing the direct binding of OsMADS29 to the promoter of *OsPUB77*. The empty constructs of pJG4‐5 and pLacZi2μ served as the negative controls. (o) Luciferase complementation imaging assay and histogram showing the interaction of OsMADS29 with the promoter of *OsPUB77* in *N. benthamiana* leaves. The pseudo‐colour bar shows the range of luminescence intensity in each image. (p) Comparison of pathway networks of GO enrichment analysis for two sets of DE genes from KO vs. 659 and OE vs. 659. The nodes represent the pathways, and the edges represent Jaccard similarity between them. All clusters can be categorised into three types: Those specific to the DE genes in ‘OE vs. 659’, those specific to the DE genes in another set of ‘KO vs. 659’, and those consensus between both sets of DE genes. (q) A proposed model for the role of *OsPUB77* and its regulator OsMADS29 in the regulation of HMRR.

To identify the key functional gene within the *qHMRR4‐2* locus, we employed a comprehensive prioritisation approach. RNA sequencing of filling seeds from phenotypically contrasting accessions (high versus low HMRR) proved highly informative. Among these, only the *LOC_Os04g49500* gene (*OsPUB77*) showed significantly differential expression in filling seeds between the two contrasting accession sets, a pattern confirmed by qRT‐PCR (Figure 1d and Figure S2). Furthermore, genetic linkage analysis showed that *OsPUB77* exhibited the highest LD with the lead SNP (with the lowest *p*‐value of *qHMRR4‐2*) (Figure S3a). Crucially, haplotype analysis provided definitive evidence: phenotyping of accessions carrying the four major *OsPUB77* haplotypes (Hap1–4) revealed that Hap4 was associated with significantly lower HMRR compared to Hap1, Hap2 or Hap3 (Figure 1e and Figure S4). Parallel haplotype‐phenotype association tests for the other candidate genes showed no significant correlation with HMRR variation (Figure S3). Leveraging 3 K genome data (Wang et al. 2018), Hap1 and Hap3 predominated in *indica*, whereas Hap2 and Hap4 clustered in *japonica* and *aus* subpopulations, respectively, with low‐HMRR Hap4 accessions concentrated in South Asia (Figure 1f,g). *OsPUB77* encodes a nucleus‐ and cytoplasm‐localised U‐box E3 ubiquitin ligase (Figure S5), a protein class implicated in regulating grain traits via cellular metabolism and protein dynamics (Wang et al. 2022). Collectively, this convergence of multi‐layered evidence, combined with functional exclusion of neighbouring genes, conclusively establishes *OsPUB77* as the key functional gene at this locus.

We established transgenic lines, comprising two gene knockout lines (KO‐1 and KO‐2) and two gene overexpression lines (OE‐7 and OE‐9), to validate the role of *OsPUB77* in regulating HMRR (Figures S6 and S7). Remarkably, both KO and OE lines exhibited decreased HMRR, accompanied by increased percentage of grains with chalkiness (PGWC) and the degree of endosperm chalkiness (DEC) (Figure 1h–k). Additionally, starch content was reduced, and the thousand‐grain weight decreased in transgenic lines at filling and ripening stages (Figure 1l,m). In contrast, no significant differences were observed in protein content, grain perimeter, grain length, grain width, grain length‐width ratio or amylose content between transgenic lines and the wild type (Figure S8). Reduced starch accumulation can increase grain susceptibility to breakage during processing, thereby leading to declined HMRR (Ma et al. 2023). Collectively, both knockout and overexpression of *OsPUB77* negatively impact HMRR, indicating that the precise regulation of *OsPUB77* expression is related to HMRR.

Given that both knockout and overexpression of *OsPUB77* reduce HMRR, we investigated upstream regulators. OsMADS29, a well‐characterised grain development regulator (Nayar et al. 2013), was identified as a direct transcriptional repressor that binds to the *OsPUB77* promoter (Table S5). This interaction was demonstrated by yeast one‐hybrid (Figure 1n), and luciferase complementation imaging assays further confirmed that OsMADS29 binding to the *OsPUB77* promoter directly represses its transcriptional activity (Figure 1o). The relative expression of *OsPUB77* peaked in the filling seeds at the 10th day after flowering (Figure S9), prompting us to perform RNA‐sequencing at the same time point. The number of differentially expressed genes (DEGs) between KO and OE lines was smaller than that between transgenic lines and wild‐type (Figure S10). This suggests that dysregulation in either direction disrupts a common set of core pathways. Indeed, GO enrichment showed that DEGs in both KO and OE lines converged on overlapping metabolic networks essential for starch biosynthesis and grain filling (Figure 1p). We propose that OsMADS29‐mediated repression fine‐tunes *OsPUB77* expression to maintain metabolic equilibrium (Figure 1q). The transcriptional repression by OsMADS29 (Figure 1n,o) likely prevents excessive *OsPUB77* expression and the attendant over‐ubiquitination of target proteins, which could disrupt the starch synthesis pathway. Optimal *OsPUB77* activity ensures starch accumulation and endosperm integrity, preventing chalkiness and grain breakage. Both insufficient and excessive *OsPUB77* disrupt starch deposition, reducing HMRR.

In conclusion, our study establishes the OsMADS29‐*OsPUB77* module as a critical rheostat for HMRR, in which precise control of *OsPUB77* safeguards starch homeostasis. This axis provides an actionable target for improving rice milling yield through precision breeding.

## Funding

This work was supported by the National Natural Science Foundation of China (32272085), Scientific and Technological Plan of Guangzhou (2024A04J7152), Special Funding for the Construction of the High‐Level Academy of Agricultural Sciences (NYQS202606, R2026PY‐BJ001), Basic and Applied Basic Research Foundation of Guangdong Province (2025A1515012969), Elite Rice Plan of GDRRI (2024YG07) and Guangdong Key Laboratory of Rice Science and Technology (2023B1212060042).

## Conflicts of Interest

The authors declare no conflicts of interest.

## Supporting information


**Data S1:** pbi70603‐sup‐0001‐DataS1.docx.

**Tables S1–S6:** pbi70603‐sup‐0002‐TablesS1‐S6.xlsx.

## Data Availability

The raw sequencing data have been submitted to the NGDC database (https://ngdc.cncb.ac.cn/bioproject/) under BioProject number PRJCA026257. More details were available in Supporting Information files.
