# Supplementary material for: The Rice E3 Ubiquitin Ligase Gene OsPUB77 Regulates Head Milled Rice Rate by Affecting Grain Starch Accumulation
Source: Plant Biotechnol J. 2026 Feb 19;24(6):3739–41. doi: 10.1111/pbi.70603 (PMC13205682; doi:10.1111/pbi.70603)
Supplement: Supplementary file 1 — Data S1: pbi70603‐sup‐0001‐DataS1.docx. [file PBI-24-3739-s001.docx]

**The rice E3 ubiquitin ligase gene *OsPUB77* regulates head milled rice rate by affecting grain starch accumulation**

Shuai Nie^†^, Luo Chen^†^, Leilei Kong^†^, Minhua Zheng, Jingfang Dong, Song Bai, Dilin Liu, Shaohong Zhang, Hao Chen, Haifei Hu, Xin Luan^*^, Junliang Zhao^*^, Wu Yang^*^

Rice Research Institute, Guangdong Academy of Agricultural Sciences; Guangdong Key Laboratory of New Technology in Rice Breeding; Guangdong Rice Engineering Laboratory; Key Laboratory of Genetics and Breeding of High Quality Rice in Southern China (Co-construction by Ministry and Province), Ministry of Agriculture and Rural Affairs, Guangzhou 510640, *P*. *R*. China

^*^ Correspondence: Tel +86-020-85161043; fax +86-020-85161043; email yangwu@gdaas.cn.

^†^ These authors contributed equally to this work.

**Supplemental Materials & Methods**

**Plant materials and growing conditions**

The 300 rice accessions selected from the RDP2 were used for phenotypic evaluation and genome-wide association study (GWAS) in this study (**Table S1**) (McCouch et al. 2016). They were planted in the second cropping season of 2016 and 2018, at the experimental site of Guangzhou (2016GZ) and Yangjiang (2018YJ) in China, respectively. The sowing and transplanting dates varied with the local planting season depending on the test environment. Management practices of local farmers were adopted. Eight plants in the middle row were harvested at maturity (35 days after flowering). The harvested seeds were naturally dried, stored in a storage at 15 ℃ for two months and then used for phenotypic evaluation. The moisture content of grain was about 13%.

**Phenotype evaluation**

The HMRR were measured according to the National Standards of the People’s Republic of China (GB/T 21719-2008). The harvested seeds were naturally dried, stored in a storage at 15 ℃ for two months and then used for phenotypic evaluation. The moisture content of the grains is controlled to approximately 13%. Briefly, 20 g of grains were dehulled by an electrical dehuller (JLG-III, Chengdu, China) and milled by a rice miller (JNM, Chengdu, China). The head milled rice weight (M) were measured. The HMRR were calculated as (M×100%÷20). All measurements were performed on two independent samples and the average value of each rice accession was used for GWAS.

**GWAS and QTL definition**

Briefly, SNPs were filtered using the criteria of having less than 10% of missing data and minor allele frequency (MAF) > 0.05. To maximize the inclusion of samples while ensuring complete phenotypic data, we executed a tailored approach by conducting SNP filtering and GWAS separately for two environments. The multiple loci mixed model (MLMM) implemented in GAPIT (Wang and Zhang 2021) was used for GWAS. In this model, the first three principal components were included as fixed effects, and the kinship matrix was incorporated as a random effect to account for population structure and genetic relatedness. Manhattan and QQ plots were produced by R-package CMplot (Yin et al. 2021). The linkage disequilibrium (LD) and LD blocks were calculated and visualized by LDBlockShow (Dong et al. 2021). The "LD decay distance" is defined as the physical span where linkage disequilibrium reduces to half its maximum value. In this study, a 200 kb distance, computed via PopLDdecay (Zhang et al. 2019), was applied as the benchmark for the flanking region length around the target QTL. The GEC software was utilized to determine significant *p*-value thresholds, with a recommended cut-off threshold of 0.00001 for *p*-values as suggested (Li et al. 2012). A locus was qualified as a potential associated QTL if flanked by two or more significant SNPs (*p* < 0.00001) within a 200 kb radius of a significant SNP. Potential functional genes were identified within the same LD block of significant associated QTL.

**Haplotype analysis**

The 300 rice accessions were re-sequenced in our previous study (Wang et al. 2023). The *R* package geneHapR was utilized to compute and illustrate the frequency distribution of haplotypes across different regions of the world (Zhang et al. 2023). To screen for candidate genes within the qHMRR4-2 region, we extracted all variants located within 2 kb upstream and downstream of each of the 31 genes in this interval. Using PLINK (Purcell et al. 2007), we then calculated inter-variant allele count squared correlations (a measure of linkage) between each gene and the lead GWAS SNP.

**Real-time PCR analysis**

RNA reverse transcription reactions were conducted using the PrimeScript TM RT reagent kit (Takara, Dalian, Liaoning, China). The qRT-PCR analysis was performed using the BioRad CFX 96 systemqRT-PCR (Pleasanton, USA). The primer sequences for qRT-PCR of *LOC_Os04g49500* (*OsPUB77*) are qOsPUB77-F and qOsPUB77-R. The eEF1α was used as the normalized gene for mRNA. All reactions were repeated thrice.

**Development of *OsPUB77* knockout and** **overexpression lines**

*OsPUB77* knockout lines were created using CRISPR/Cas9 editing. The sequences of two small guide RNA were respectively 5’-GCCGGAGGGTTACTGTCAGG CGG-3’ and 5’-GACCGCCACACGGAGGTCAT CGG-3’. The amplification primers used to identify positive knockout plants were pcrPUB77-F and pcrPUB77-R. For the overexpression lines, the coding sequence of *Os**PUB77* was amplified from the *indica* accession (No. 659) and subcloned into the overexpression vector under control of the DXCP35 promoter, which was identified as an endosperm-specific promoter (Yan Y and YJ. 2014). The constructed plasmids were transferred into the *indica* accession (No. 659) via Agrobacterium tumefaciens EHA105 by an *Agrobacterium-mediated* genetic transformation approach.

**RNA-sequencing and data analysis**

For RNA-sequencing, three accessions with high HMRR (No. 539, 645 and 659) and three accessions with low HMRR (No. 452, 523 and 651) were selected based on haplotype analysis. The spikes of the six accessions were sampled at 14 days after flowering. To study the pathway of *OsPUB77* in regulating HMRR, three independent spikes from wild type, *OsPUB77* knockout and overexpression lines were sampled on the 10^th^ day after flowering. Total RNA was extracted using Trizol reagent (Takara, Dalian, Liaoning, China). RNA-sequencing was conducted by Annoroad Gene Technology (Beijing, China).

The HISAT2-featureCounts-DEseq2 pipeline was used for data analysis with default parameters (Kim et al. 2019; Liao et al. 2014; Love et al. 2014). The Nipponbare genome (MSU v7.0) was used as a reference for reads mapping (Kawahara et al. 2013). Then differentially expressed genes between two contrasting accessions were identified according to the criteria of adjusted t-test *p*-value ＜ 0.05 and fold change ≥ 2 or ≤ 0.5. GO enrichment analysis were performed with an R package clusterProfiler (Yu et al. 2012). Network comparisons of enriched GO pathways were obtained and visualized by an R package aPEAR (Kerseviciute and Gordevicius 2023).

**Subcellular localization of *OsPUB77***

To elucidate the localization of *OsPUB77*, we employed a constructed OsPUB77-GFP fusion protein, which was transiently expressed in rice protoplasts under the control of the CaMV 35S promoter. The GFP fusion protein was analyzed by a confocal laser scanning microscope (FV1000 OLYMPUS) with a 488-nm exciting wavelength. The exciting wavelength for chloroplast 640-nm. SV40 was used as the nuclear marker (Kalderon et al. 1984).

**Determination of starch and protein content**

The grains were harvested at different times during the filling period, dried to the constant weight with approximately 13% moisture content. 20 g of grains were first de-husked using a huller (JLGII, Chengdu, China) and subsequently polished with a rice polisher (JNM, Chengdu, China). The polished grains were then ground into flour using a grinder (LM3100, Stockholm, Sweden) and passed through a 100-mesh sieve. The protein content was determined according to the national standard of food safety in China (GB 5009.5-2016). The starch content was determined according to the reported study (Deng et al. 2021). Amylose content was pretreated following the national standard (GB/T 15683-2008, China) and measured using an automatic amylose analyzer (Futura-II, Frepillon, France). Amylopectin content was obtained by subtracting the content of amylose from the total starch content.

**Protein-DNA binding analyses**

For Y1H assay, the full-length coding sequence of OsMADS29 was synthesized by the whole sequence of GenScript Biotech Corporation and then cloned into the pJG4-5 vector at the EcoRI and XhoI restriction sites. The promoter of *OsPUB77* was amplified using the primers of 24EV35-F and 24EV35-R (**Table S6**) and then cloned into the pLacZi2μ vector at the EcoRI and XhoI restriction sites. The plasmids of pJG4-5-OsMADS29 and pLacZi2μ-Os*PUB77*-pro were co-transformed into the yeast strain EGY48. The yeast transformation and analysis methods referred to the published study (Liu et al. 2021). The positive control could appear blue on the chromogenic medium plate, and the negative control was white.

For the luciferase-based transient transcriptional activity assay, the full-length coding sequence of OsMADS29 was cloned into the pGreenⅡ62-SK vector at the KpnI and BamHI restriction sites. The promoter of *OsPUB77* was amplified using the primers of 24EV77-F and 24EV77-R (**Table S6**) and then cloned into the pGreenⅡ 0800-LUC vector at the KpnI and BamHI restriction sites. The plasmids of the four groups (pGreenⅡ62-SK-OsMADS29 + pGreenⅡ 0800-LUC-Os*PUB77*-pro, pGreenⅡ62-SK-OsMADS29 + pGreenⅡ 0800-LUC, pGreenⅡ62-SK + pGreenⅡ 0800-LUC-Os*PUB77*-pro, pGreenⅡ62-SK + pGreenⅡ 0800-LUC) were respectively transformed into *A. tumefaciens* strain *GV3101*. The transformation and analysis methods referred to the published study (Li et al. 2022). The luciferase signals were detected by a Chemiluminescence Imaging System (Tanon 5200).

**References**

Deng F, Li Q, Chen H, Zeng Y, Li B, Zhong X, Wang L, Ren W (2021) Relationship between chalkiness and the structural and thermal properties of rice starch after shading during grain-filling stage. Carbohydrate Polymers 252:117212. doi:10.1016/j.carbpol.2020.117212

Dong SS, He WM, Ji JJ, Zhang C, Guo Y, Yang TL (2021) LDBlockShow: a fast and convenient tool for visualizing linkage disequilibrium and haplotype blocks based on variant call format files. Briefings in Bioinformatics 22 (4). doi:10.1093/bib/bbaa227

Kalderon D, Richardson WD, Markham AF, Smith AE (1984) Sequence requirements for nuclear location of simian virus 40 large-T antigen. Nature 311 (5981):33-38. doi:10.1038/311033a0

Kawahara Y, de la Bastide M, Hamilton JP, Kanamori H, McCombie WR, Ouyang S, Schwartz DC, Tanaka T, Wu J, Zhou S, Childs KL, Davidson RM, Lin H, Quesada-Ocampo L, Vaillancourt B, Sakai H, Lee SS, Kim J, Numa H, Itoh T, Buell CR, Matsumoto T (2013) Improvement of the *Oryza sativa* Nipponbare reference genome using next generation sequence and optical map data. Rice (N Y) 6 (1):4. doi:10.1186/1939-8433-6-4

Kerseviciute I, Gordevicius J (2023) aPEAR: an R package for autonomous visualization of pathway enrichment networks. Bioinformatics 39 (11). doi:10.1093/bioinformatics/btad672

Kim D, Paggi JM, Park C, Bennett C, Salzberg SL (2019) Graph-based genome alignment and genotyping with HISAT2 and HISAT-genotype. Nature Biotechnology 37 (8):907-915. doi:10.1038/s41587-019-0201-4

Li MX, Yeung JM, Cherny SS, Sham PC (2012) Evaluating the effective numbers of independent tests and significant *p*-value thresholds in commercial genotyping arrays and public imputation reference datasets. Human Genetics 131 (5):747-756. doi:10.1007/s00439-011-1118-2

Li Y, He Y, Liu Z, Qin T, Wang L, Chen Z, Zhang B, Zhang H, Li H, Liu L, Zhang J, Yuan W (2022) OsSPL14 acts upstream of *OsPIN1b* and *PILS6b* to modulate axillary bud outgrowth by fine-tuning auxin transport in rice. The Plant Journal 111 (4):1167-1182. doi:10.1111/tpj.15884

Liao Y, Smyth GK, Shi W (2014) featureCounts: an efficient general purpose program for assigning sequence reads to genomic features. Bioinformatics 30 (7):923-930. doi:10.1093/bioinformatics/btt656

Liu Y, Wu G, Zhao Y, Wang HH, Dai Z, Xue W, Yang J, Wei H, Shen R, Wang H (2021) DWARF53 interacts with transcription factors UB2/UB3/TSH4 to regulate maize tillering and tassel branching. Plant Physiology 187 (2):947-962. doi:10.1093/plphys/kiab259

Love MI, Huber W, Anders S (2014) Moderated estimation of fold change and dispersion for RNA-seq data with DESeq2. Genome Biology 15 (12):550. doi:10.1186/s13059-014-0550-8

McCouch SR, Wright MH, Tung CW, Maron LG, McNally KL, Fitzgerald M, Singh N, DeClerck G, Agosto-Perez F, Korniliev P, Greenberg AJ, Naredo ME, Mercado SM, Harrington SE, Shi Y, Branchini DA, Kuser-Falcão PR, Leung H, Ebana K, Yano M, Eizenga G, McClung A, Mezey J (2016) Open access resources for genome-wide association mapping in rice. Nature Communications 7:10532. doi:10.1038/ncomms10532

Purcell S, Neale B, Todd-Brown K, Thomas L, Ferreira MA, Bender D, Maller J, Sklar P, de Bakker PI, Daly MJ, Sham PC (2007) PLINK: a tool set for whole-genome association and population-based linkage analyses. American Journal of Human Genetics 81(3):559-75. doi: 10.1086/519795.

Wang J, Yang W, Zhang S, Hu H, Yuan Y, Dong J, Chen L, Ma Y, Yang T, Zhou L, Chen J, Liu B, Li C, Edwards D, Zhao J (2023) A pangenome analysis pipeline provides insights into functional gene identification in rice. Genome Biology 24 (1):19. doi:10.1186/s13059-023-02861-9

Wang J, Zhang Z (2021) GAPIT Version 3: Boosting Power and Accuracy for Genomic Association and Prediction. Genomics Proteomics Bioinformatics 19 (4):629-640. doi:10.1016/j.gpb.2021.08.005

Yan Y, YJ. L (2014) Cloning and functional indentification of an endosperm-specific promoter DXCP35 from rice. Jorunal of Huazhong Agricultural University 33 (05):15-20. doi:10.13300/j.cnki.hnlkxb.2014.05.027

Yin L, Zhang H, Tang Z, Xu J, Yin D, Zhang Z, Yuan X, Zhu M, Zhao S, Li X, Liu X (2021) rMVP: A memory-efficient, visualization-enhanced, and parallel-accelerated tool for genome-wide association study. Genomics Proteomics Bioinformatics 19 (4):619-628. doi:10.1016/j.gpb.2020.10.007

Yu G, Wang LG, Han Y, He QY (2012) clusterProfiler: an R package for comparing biological themes among gene clusters. OMICS 16 (5):284-287. doi:10.1089/omi.2011.0118

Zhang C, Dong SS, Xu JY, He WM, Yang TL (2019) PopLDdecay: a fast and effective tool for linkage disequilibrium decay analysis based on variant call format files. Bioinformatics 35 (10):1786-1788. doi:10.1093/bioinformatics/bty875

Zhang R, Jia G, Diao X (2023) geneHapR: an R package for gene haplotypic statistics and visualization. BMC Bioinformatics 24 (1):199. doi:10.1186/s12859-023-05318-9

**Supplemental Figures**


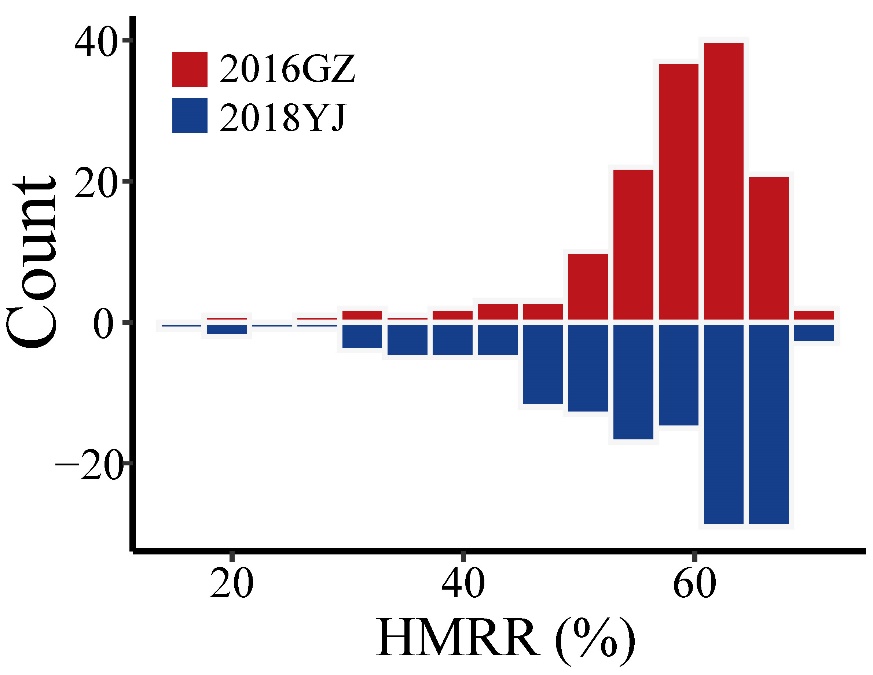


**Figure S1**. Phenotypic distributions of HMRR in two environments. The 2016GZ and 2018YJ represent the two environments.


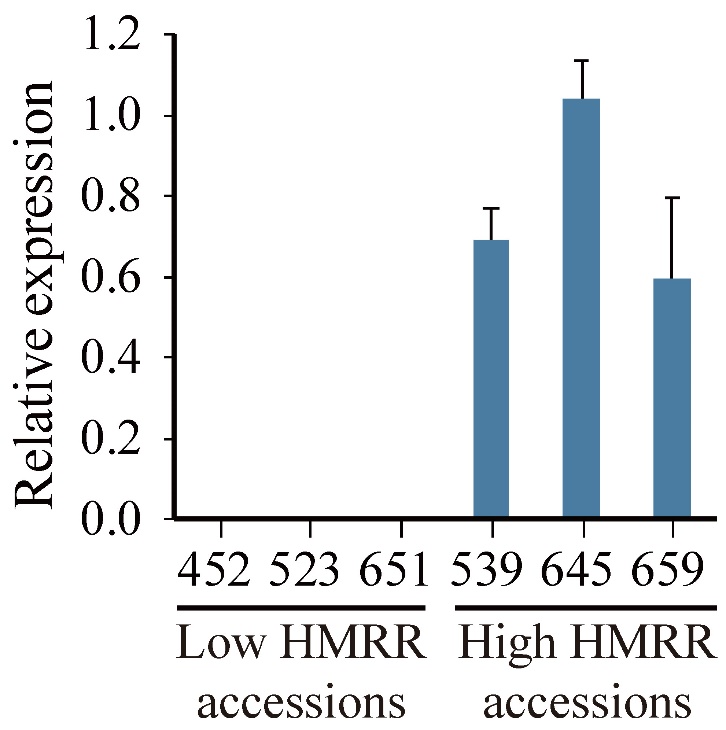


**Figure S2**. Expression analysis of *LOC_Os04g49500* for *qHMRR4-2*. The spikes were sampled on the 14^th^ day after flowering of three accessions with high HMRR (accessions 539, 654 and 659) and three accessions with low HMRR (accessions 452, 523 and 651) were selected based on haplotype analysis.


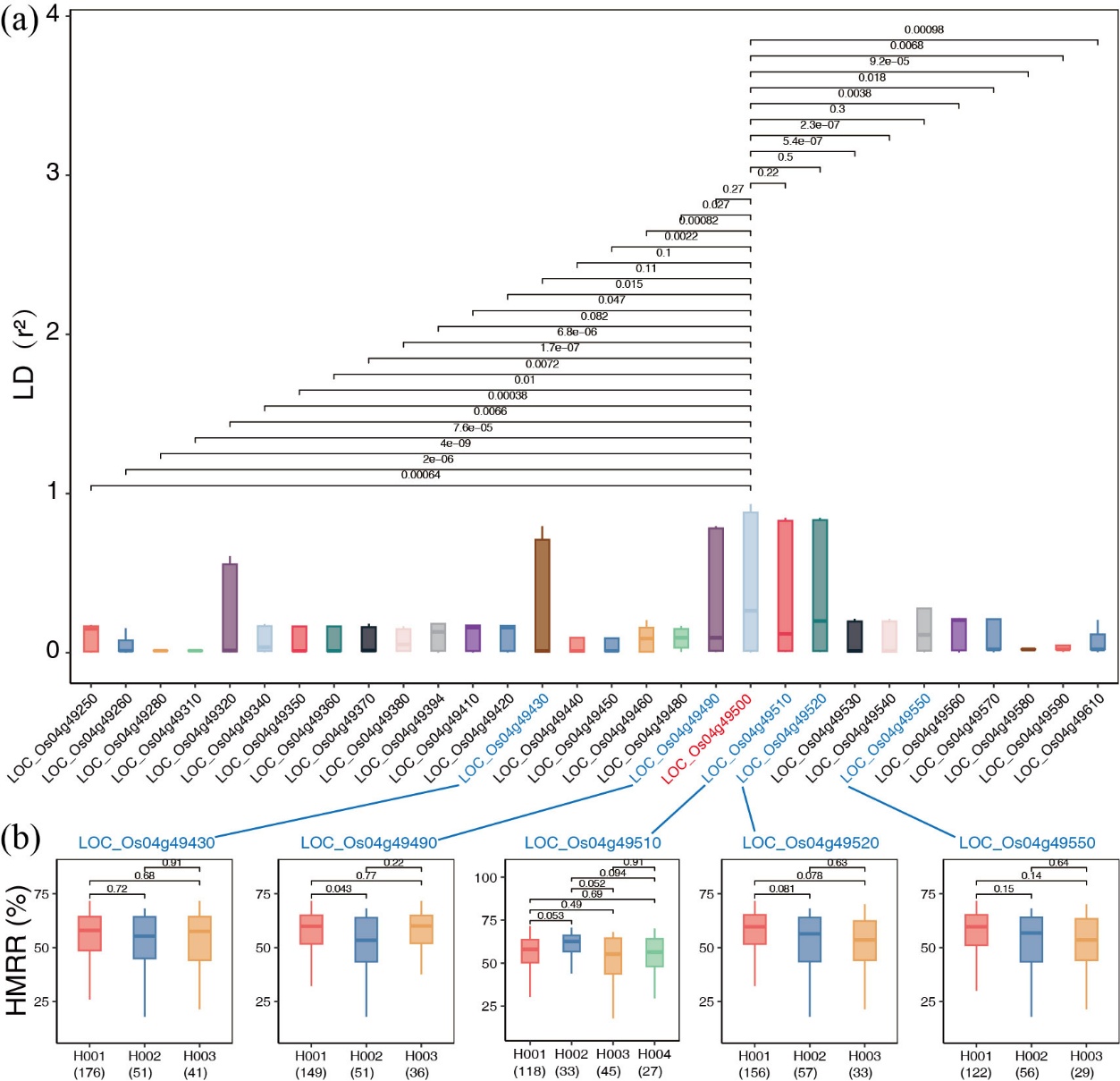


**Figure S3**. Genetic validation of *LOC_Os04g49500* (*OsPUB77*) as the functional candidate gene in *qHMRR4-2*.​(a)​Linkage disequilibrium (LD) analysis between the lead SNP and 31 candidate genes in *qHMRR4-2*. *LOC_Os04g49500* shows the highest LD (r²) with the lead GWAS SNP (marked in red). Five other genes (marked in blue) exhibited r² values statistically indistinguishable from *LOC_Os04g49500* (*t*-test, *p* > 0.05). (b)​Haplotype-based phenotypic validation of five candidate genes. Boxplots show head milled rice rate (HMRR) distributions across haplotypes of each gene. No significant differences (*t*-test, *p* > 0.05) were observed for any gene, excluding them as functional candidates.


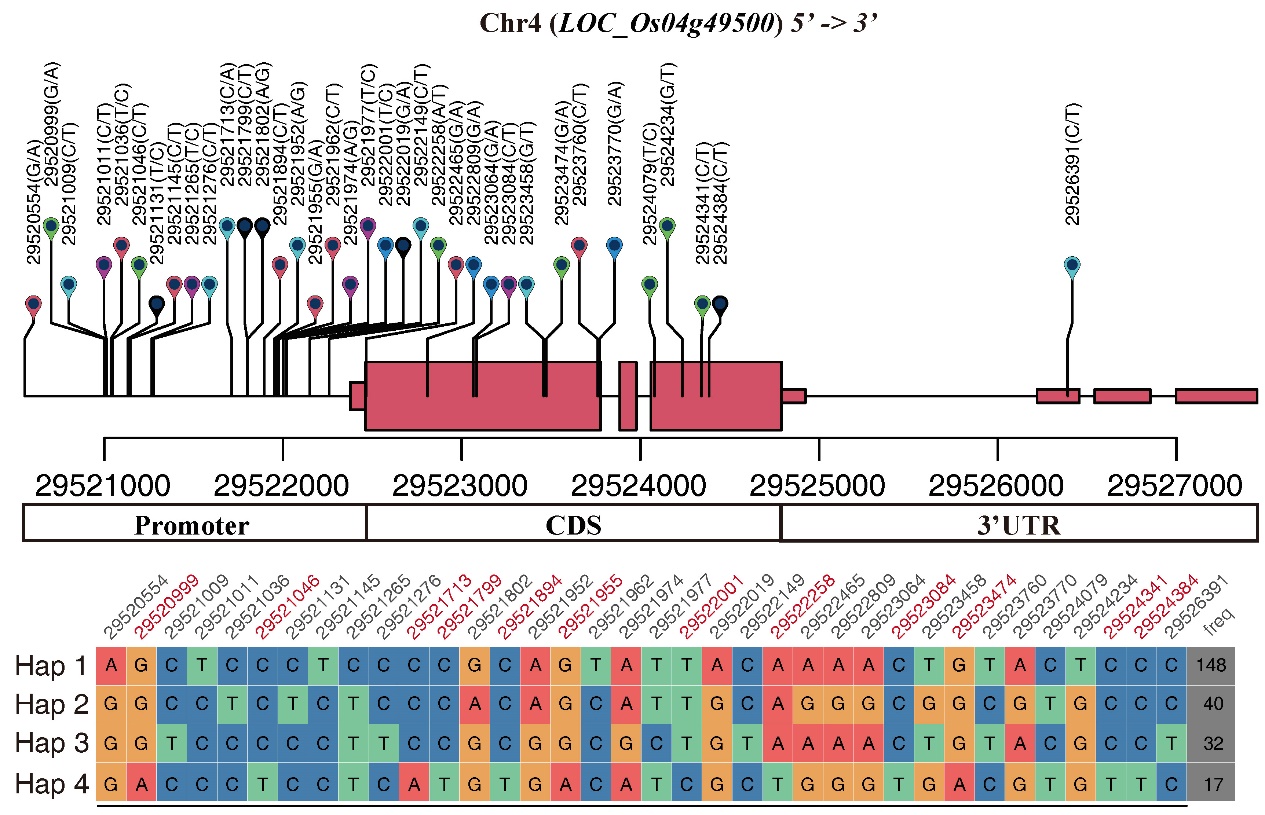


**Figure S4**. Four haplotypes of *OsPUB77* were identified from the whole genome re-sequencing data of 300 rice accessions used in this work.


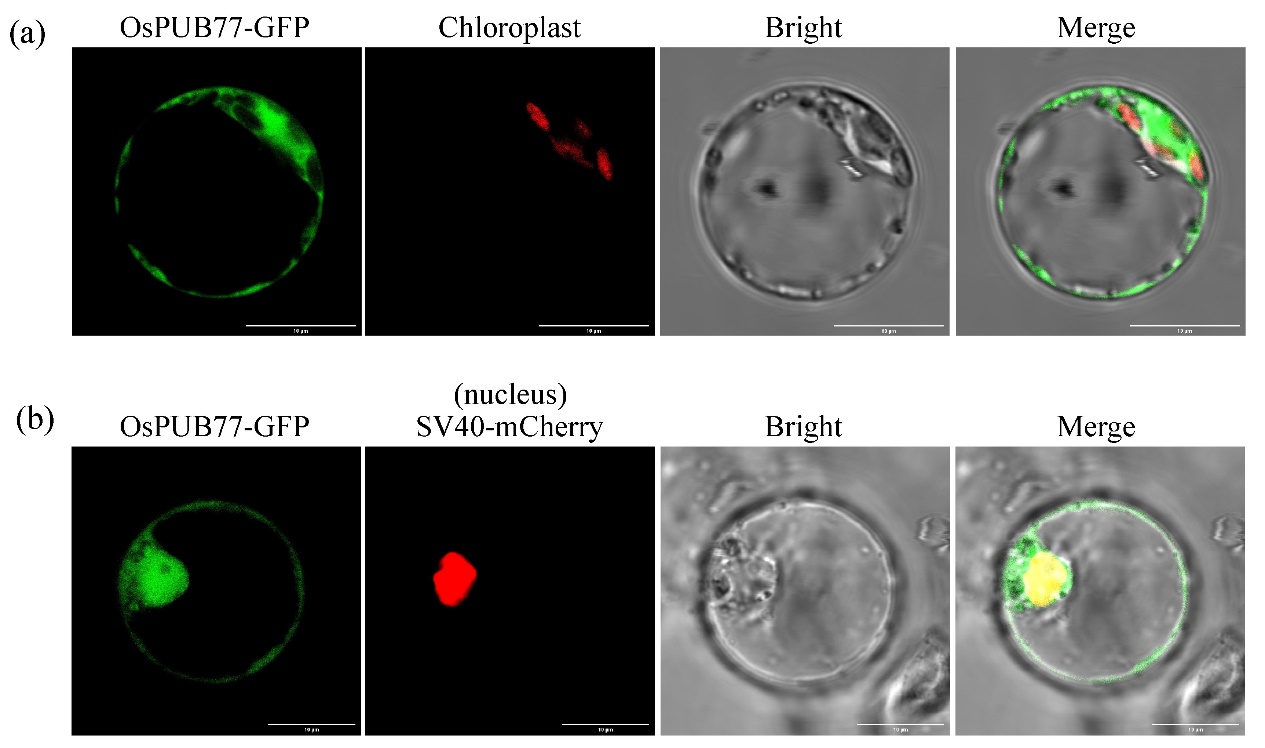


**Figure S5**. Subcellular localization of *OsPUB77.*


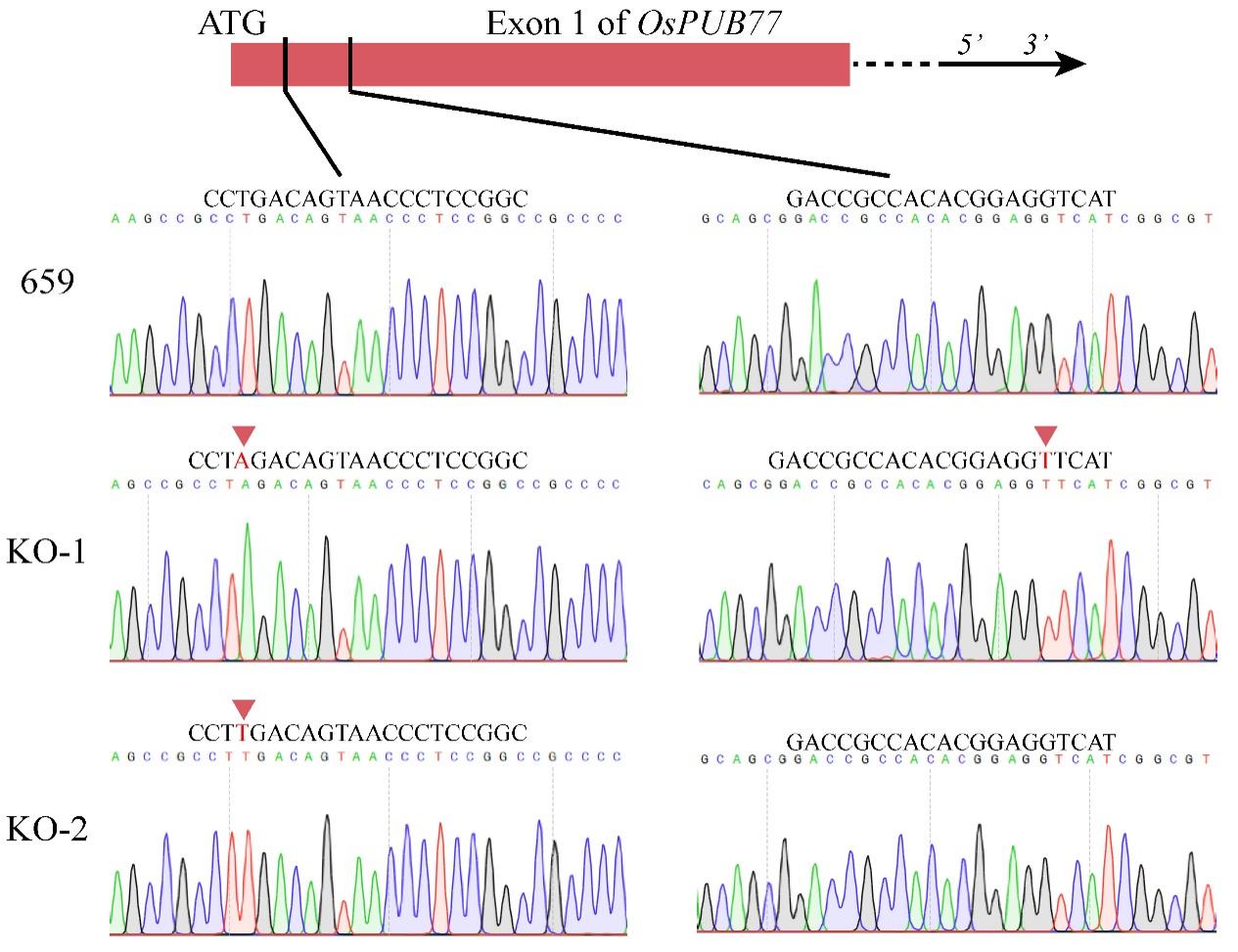


**Figure S6**. Sequences of CRISPR-*OsPUB77* alleles in KO lines. sgRNA target sequences (*OsPUB77*-PS1 and *OsPUB77*-PS2) are showed, with mutation positions indicated by red arrows.


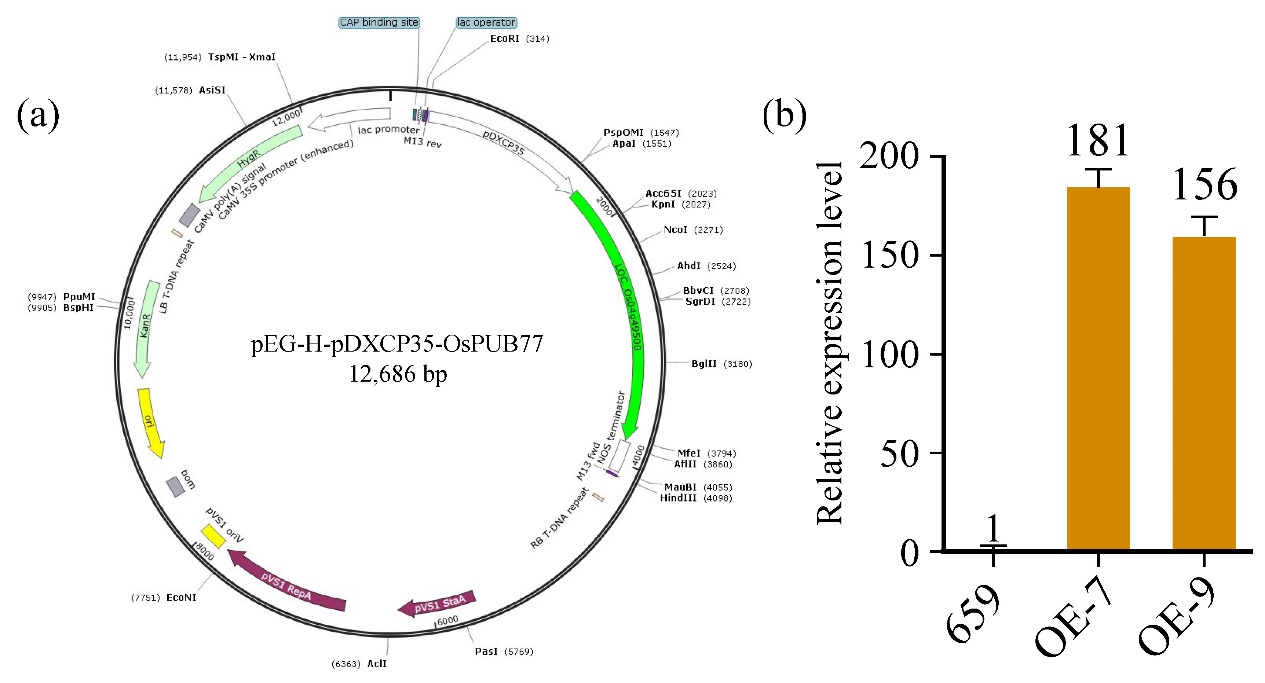


**Figure S7**. *OsPUB77* overexpression lines. (a) Overexpression vector. (b) Relative expression levels of *OsPUB77* in overexpression lines. The transcript level of *OsPUB77* in wild type (659) was used as control and set to a value of 1.


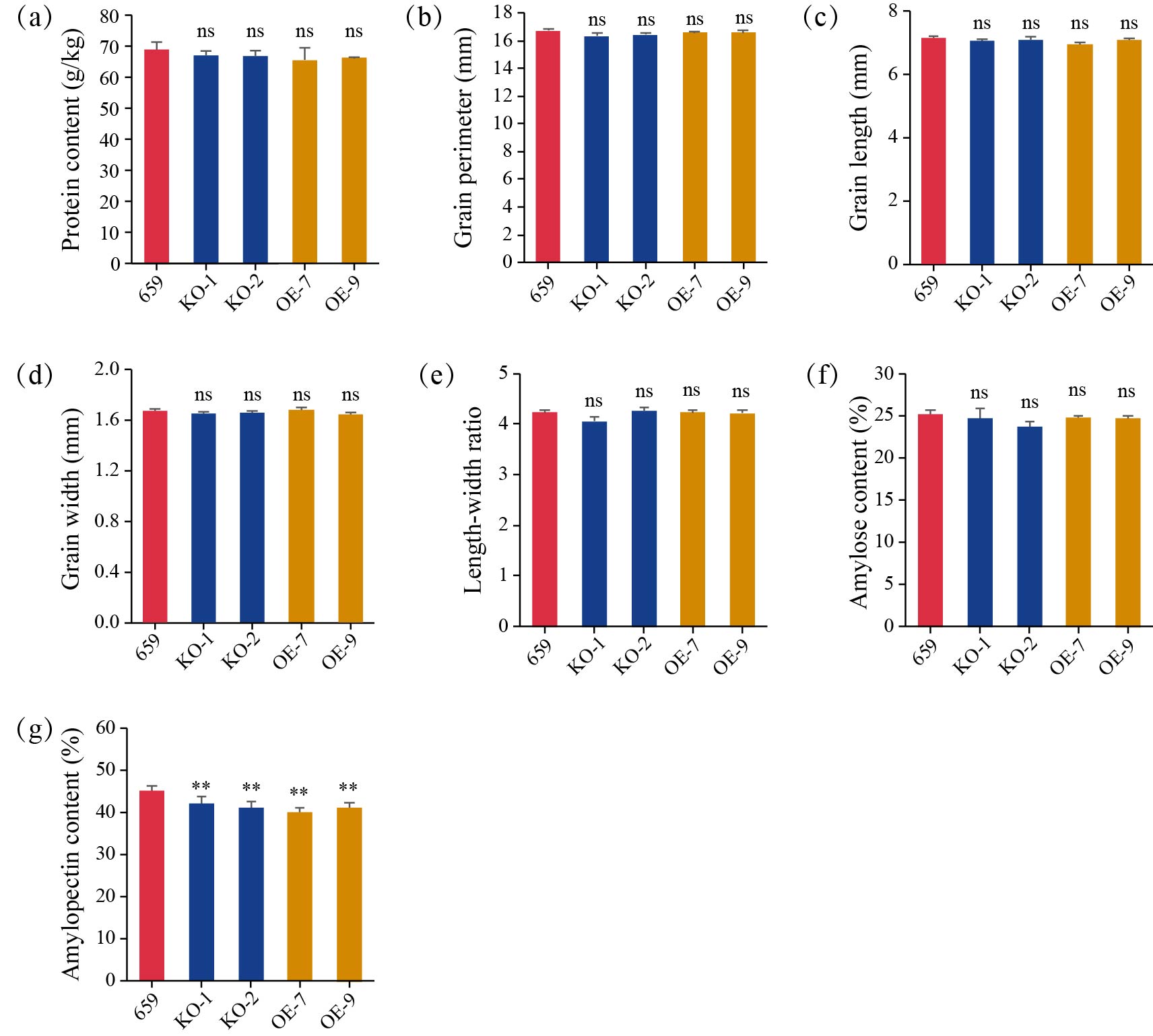


**Figure S8**. Comparisons of grain traits in wild type and transgenic lines. (a)-(g) represent protein content, grain perimeter, grain length, grain width, length-width ratio, amylose content, and amylopectin content, respectively. Two biological replicates were measured. Means comparisons employed two-tailed *t*-tests (*, *P* < 0.05; **, *P* < 0.01; ns, no significance).


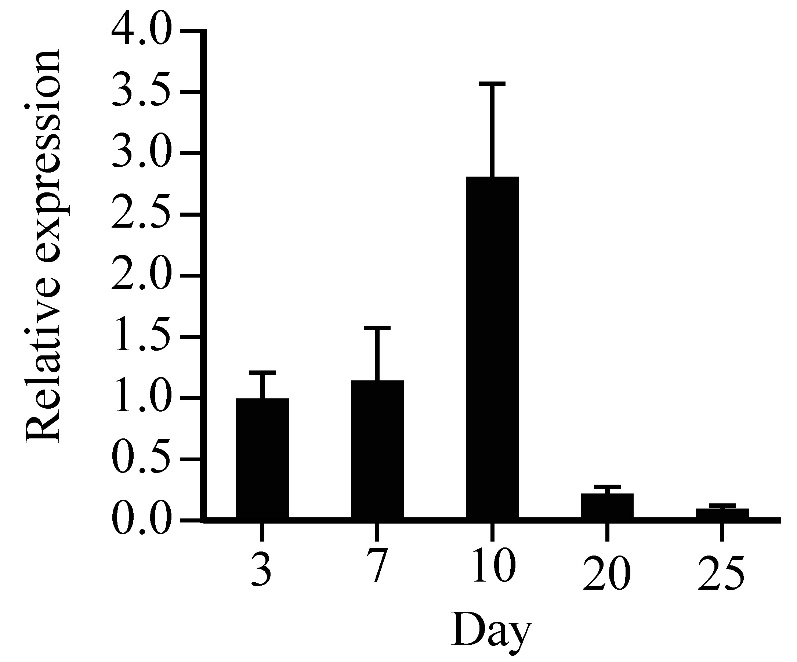


**Figure S9**. Relative expression of *OsPUB77* in filling seeds during grain filling. The expression level of *OsPUB77* at 3^th^ day after flowering was used as control and set to a value of 1.


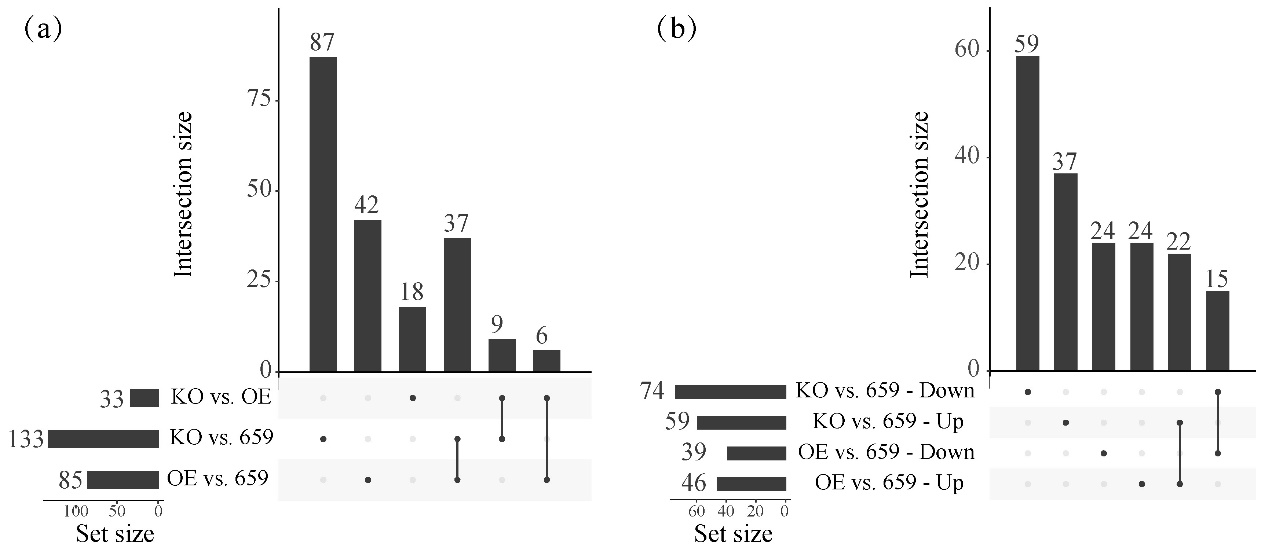


**Figure S10**. UpSet plots of differentially expressed genes (DEGs) between the KO or OE lines with the wild type (No. 659). Black dots show gene set intersections (connected) or unique sets (single). Vertical bars indicate intersection sizes; horizontal bars show total DEGs per comparison. (a) Overlap of all DEGs, showing fewer genes in the KO vs. OE comparison (33) than in comparisons with the wild type. (b) Overlap of up- and down-regulated DEGs for KO vs. 659 and OE vs. 659.
